# An Address, Introductory to the Eighth Regular Summer Course of Lectures in the Atlanta Medical College

**Published:** 1866-07

**Authors:** S. H. Stout

**Affiliations:** Professor of Surgical and Pathological Anatomy


### ARTICLE II.

*An Address, introductory to the Eighth Regular Summer Course of Lectures in the Atlanta Medical College.* By S. H. Stout, M. D., Professor of Surgical and Pathological Anatomy.

### CORRESPONDENCE.

Atlanta, May 18th, 1866.

Prof. S. H. Stout:

*Dear Sir:*—The members of the present class of the Atlanta Medical College, having been much pleased with the able Introductory Address delivered by you, and believing that a reviewal of it would be both interesting and beneficial, have appointed the undersigned a committee to request of you a copy for publication.

Hoping you will comply with our request, and assuring you of the high esteem with which you are regarded by the class, we remain,

With much respect, yours.

Robt. S. Cameron, of Ga.,
F. D. Nabers, of Ala.,
C. O. O. Roberts, of Fla.,
M. D. Sterrett, of Texas,
A. E. Fant, of S. C.,
R. P. Spencer, of Va.,
G. W. Anderson, of Miss.,
Committee.

---

Atlanta, Ga., May 21st, 1866.

Gentlemen:

I cannot refuse to comply with your request contained in yours of the 18th inst., and herewith hand you a copy of the address alluded to.

Grateful for the expression of the kind regard of the class whom you represent,

I am respectfully your ob't sv't,

S. H. Stout.

To Messrs. Cameron, Nabers, Roberts, Sterrett, Fant, Spencer, Anderson, Committee.

### ADDRESS.

*Gentlemen of the Medical Class:*

Pledging their unremitting attention to their duties as your teachers and friendly advisers, in the name of the Faculty of the Atlanta Medical College, I bid you *welcome.*

For and on behalf of the good citizens of Atlanta, I assure you of their sympathies and kind wishes for your success in the pursuit of the object of your sojourning among them.

The many landmarks and associations suggestive of the past, which here attract the attention, are combined with many incidents and facts which forcibly remind us of present duties, and indicate that our future is not altogether hopeless of a return to happiness and prosperity.

That pile of ruins, those blackened walls, that line of rifle-pits, those redoubts, those fields, beneath which lie the Federal and Confederate dead, every living man and woman, and almost every child you see, have a tale to tell of devastation and desolation, of marches and countermarches, of battles fought, of victory and defeat, of exile, of hopes disappointed, of sorrow, of terror and despair.

But the busy, active throng of merchants, traders, manufacturers, mechanics and laborers—the whistle of the locomotive, the hum of machinery, the sound of axe and hammer, and the moving mass of merchandise and building material—give earnest that the recuperative energies of the people of the subjugated South are not totally destroyed; that there is "life in the old land yet;" that we can adapt ourselves to our changed circumstances, and from the *debris* of our fortunes and of society, revive our material interests; rebuild, with some additions and changes of plan, our social structure; re-open our institutions of learning; restore order and embark in enterprises promising honorable remuneration for labor and toil.

"History is philosophy teaching by example." And such a history as has been ours during the last five years, cannot fail to exercise an influence for good upon the temper and future conduct of our people.

The very disasters they have met with, the losses they have experienced, the earnestness of their resistance, and the realization of their worst fears, impress them that there are yet duties to be performed—new and weighty responsibilities to meet.

Never, in the history of any people, has society experienced such convulsions. Never was there a greater necessity for wisdom in council, integrity of purpose and honor in the performance of every duty. The history of the Southern people gives assurance that they are frank and trustworthy. Their present conduct indicates their claim to this high character.

How each and every one may meet the obligations of the hour, is exercising the thoughts of every conscientious and intelligent man and woman in the land.

To the politicians and statesmen the people have committed the business of untangling the complicated politics of the country, and these are now intensely occupied with their task. The merchant, mechanic, and agriculturist, having each in his sphere appropriate duties to perform and responsibilities to meet, are busily engaged in restoring trade, inaugurating new enterprises, developing new resources, enhancing the value of raw material by industry and skill, and persuading the soil to yield the products so necessary to our comfort and prosperity. Thus quietly, unobtrusively, this earnest people are struggling to arise self-supporting from the ashes of their ruined fortunes and institutions.

Never has there been exhibited such moral heroism; such quietude after a stupendous war—after such ruin and devastation. The epoch in which we live will, ages hence, be celebrated; and if we succeed in securing a return to prosperous peace, the wisdom of our policy will be as much admired as the exploits of our heroes.

Having lost our cause by the verdict of the tribunal of arms, to which we appealed, like a family bereft of its head, we have returned to our homes, after performing the funeral obsequies of the Confederate States Government, when the last gun was fired, and the last cannon roared.

We would be dishonest to profess that we regret not our loss.

We are honest in accepting the situation and in rejoicing that bloodshed has ceased, and that we are again permitted to cultivate the arts of peace.

Of the resurrection of the dead cause, men of common sense have no hope. For the age of miracles has passed, and the stern realities of the situation forbid the vain indulgence of the imagination.

We do truly mourn for our missing heroes dead. Though no proud monuments may mark the spots where rest the brave,

> "By fairy hands their knell is rung,
> By forms unseen their dirge is sung;
> There honor comes, a pilgrim gray,
> To bless the turf that wraps their clay."

And while memory lasts or thought endures, the true people of the South—and these are they who will not violate their obligations—will ever love to dwell with pride upon their history and their exploits and with mournful sadness upon their sacrifices.

Having accepted the situation, yet ignoring none of the nobler impulses of our nature, with the realities of the present we are now engaged. On this occasion it is, therefore, proposed briefly, and in the plainest language, to call attention to the duties and obligations of educated and scientific men, and some of the circumstances which encourage them to prompt and energetic action. This is not an appropriate time—though ordinarily the occasion might justify and demand it—to indulge in a disquisition upon some theoretical subject. But we must now be practical. We must calmly view the situation, and each and every one of us must appreciate his duty and honestly attempt its performance.

After the devastations of the war educators and votaries of science have much to do in resuscitating old institutions of learning and erecting new ones; in improving the physical condition of the people; in developing the resources, animal, mineral and vegetable, of the country; in diffusing knowledge; in promoting science; in giving direction to plans and schemes for social improvement; in fostering the energies and encouraging the progressive spirit of the people.

Never was there a period when this people were so well prepared to listen intelligently to propositions for their individual and social improvement, if made by those in whom they trust. Never were a people so habituated to benevolent action or so ready to heartily engage in any good work which many receive the approbation of their judgment.

They have been to school, and under that inexorable teacher, Necessity, they have become more than ever enlightened as to the real value of science and thorough education ; and their hearts have been purified, as it were, by fire.

The youth of 1861 are the men of 1866. Circumstances have afforded them opportunities for mental exercise, have enlarged their views, and furnished educational facilities, which, perhaps, no soldiers ever before enjoyed.

For, notwithstanding the theoretical inequality of the private soldier and his officer, the armies of the South were, in camp and upon the march, great social gatherings in which mind met mind, and freedom of discussion and independence of thought were never repressed.

Thus every soldier of honorable impulses and good mental capacity has been educated into a traveled gentleman of enlarged and liberal views, of determination of purpose, of fortitude to endure hardships, of courage to face dangers, and earnestness in the performance of duty.

Such are the men who are to give tone to society among us. Such are the men whose judgments are to be appealed to, and who are to sustain every good work proposed. With them the arts of the demagogue are powerless of harm. They know, better than their predecessors, the value of scientific skill; and such men will not encourage mountebanks or be indifferent to learning and social progress.

The daily habit of benevolence, indulged in, during the war, by men, women and children, by citizens and soldiers, has ennobled, elevated and softened the hearts of the people. Hence selfishness is not respectable ; and the rejoicing in each other's welfare and the promotion of each other's happiness, are daily sources of pleasure and means of employment, to an extent hitherto unknown.

And shall we fear that they will cease their good works because, forsooth, some have taken the thirty pieces of silver and are now clothed in purple and fine linen? No! The comforting recollection of duty, well and honestly performed, ensures perseverance in the practice of charity and stimulates earnestness in the meeting of obligations. They are still ready to second schemes of benevolence and improvement. The signs of the times encourage their advocacy and inauguration.

Never were the white people of the South more homogeneous than now, and their animus upon most subjects is worth preserving. It can be preserved by educating our youth at home, where natural and adventitious causes are constantly in operation to give it exercise, to develop and to cherish it. And *it is* possible, that they *may* there enjoy the light of science, the privileges of civilization, while preserving the peculiar ethnological features, mental and moral, of a people such as we have described.

Do not understand us as contending that truth is measured by a different standard among us from that which must be used by other enlightened people. From its very nature, its measure is unalterable. Yet every reflecting mind must admit that upon the animus of a people depends, to a great extent, their zeal for the investigation of truth and for its unselfish acknowledgment.

Climate and other adventitious causes, no doubt, aided in giving direction to the out-of-doors observations of Gallileo in Italy, and to the abstruse studies of Newton in England, which unitedly resulted in the revival and establishment of the Copernican theory of astronomy upon the basis of mathematical demonstration.

The true votary of science is cosmopolitan. Observing facts and deriving ideas from the savage Esquimeau, devouring the raw blubber of a seal, or from the Parisian, genteelly feasting upon chemically prepared viands and beverages, he recognizes ethnological peculiarities, which give direction, in various degrees, to investigation and impulse to effort. He knows that climate, the products of the soil, and the pursuits of a people, their interests, their traditions and the customs of the first inhabitants of the country, do, to a large extent, influence their morals and their tastes.

The conquered people of the South having passed under the yoke and conformed to the terms of the victors, are still the same generous, independent thinkers they ever were. Having won name and fame, they must more than heretofore be distinguishable by their temperament and their social customs.

The natural and artificial causes, which fostered the Puritan settlement at Plymouth, and gave prosperity to the French Huguenots at Charleston, still continue to model and shape, in part, the mental and moral features of the people of the two great sections of these United States. Immigrants into either section and their descendants, will ever continue to be affected by their influence.

From different stand-points, with different opportunities to ascertain correct premises, representative men came to opposite conclusions upon great social and political questions, and hence, the recent sacrifice of perhaps a million of lives and unnumbered woes.

Enough then has occurred and is patent to all, to prove the existence of ethnological differences between the people of the two sections, to persuade intelligent men of the folly of attempting to enforce uniformity of social customs. Without crimination and recrimination, ridicule and satire, why may not every people cherish their own desirable characteristics without demanding that others shall be like them?

If we are convinced that we possess desirable and ennobling impulses and characteristics, let us perseveringly cherish them, and as a means of cherishing them, let us ourselves direct the education of our youth, train our young men and women, and encourage their laudable enterprises.

The war decided against us on two issues and only two, viz: The supremacy of the Federal Government, and the abolition of slavery. Upon all other questions there was no contest. Therefore, our identity as a people is not lost; nor has our right to direct the education of our children been taken away.

To preserve the civilization of the Southern people, to improve it, and to make it felt in the peaceful pursuits of life, is a high and holy task incumbent on our educated and intelligent men and women.

Now is the time to inaugurate their plans and to propose their schemes. The greater the difficulties and the greater the embarrassments, the more food for thought, the more stimulus to effort, and the better the opportunity for the display of genius.

And they have much to stimulate them, even in this time of pecuniary distress and embarrassment. The perseverance of the people, their just appreciation of the good and true, the liberality of their views which engenders a proper degree of docility in the pupil, the combination of causes which tends to develop individuality and independence of thought, the very novelty of the situation, the passing away of old social elements and the advent of new ones, unite to render the task one of peculiar pleasure and responsibility as well as of immediate necessity.

No one capable of exercising influence for good, unless in danger of being dragged to the scaffold where his blood would be uselessly or unjustly spilled—no one whose example and words of counsel would promote the interests of this people, should now desert them.

The man of wealth may have it in his power to emigrate. The distinguished citizen may find employment and have honors heaped upon him in foreign parts; but the great living, toiling mass of the people must stay at home. Here they must find a field of action. Here they must rear their children. Here, too, they must be buried by the graves of their fathers. The exodus of the whole people, even if desirable, cannot take place. Here, then, should all, who can peacefully do so, remain to share a common destiny and to contribute each his mite to the common welfare.

Without dwelling upon the display of brilliant genius, which, for four years, in the field and the forum, aroused the attention of the world, when our Lee and the Johnstons, Beauregard and Bragg, Stonewall Jackson and Hood, and hosts of others were making startling history, or recalling the intellectual achievements of our representative men prior to the war; let us look behind the scenes, where, less patent to public gaze, the corps of scientific men were bidding nature to yield up her treasures and art to shape them into materiel of war. Silently, ungazetted, at their touch, mines and caverns exposed their riches, smelting furnaces and foundries, unrivalled powder mills, iron works and rolling mills arose as if by magic. These, and those triumphantly successful artificial nitre beds, and superior arms and fixed ammunition, attest the genius, skill and scientific attainments of the officers of the Nitre and Minining and Ordnance Bureaus.

They demonstrated that the Southern States contain valuable and varied resources amply sufficient for the sustenance of the people in peace as well as in war. They have but to assert their claim to the approbation of the scientific world, and that candid tribunal will acknowledge it. For they can, by their achievements in chemical manufactures, in mining and in practical [geology, prove a claim to peerage with the scientific corps of any nation. These men should not withdraw their light, but let it continue to be seen, that all may avail themselves of their assistance in diversifying their pursuits, and inagurating new enterprises.

When the shipping interests of the South become extensive, there will be need for the services of all our Maurys. When the rich veins of ore are to be traced, as soon they must be, the geologist and mineralogist will be sought for. Chemistry, civil engineering, natural history and natural philosophy must all be pressed into the service of the people in aid of the various enterprises into which they will embark. Among the scientific corps alluded to, there are many who can achieve as much in the peaceful pursuits of life as they did in war.

And what shall we say of the medical profession? What is now the duty of its members? The energy exerted, the disinterestedness exhibited by them, and their humane sympathy with the suffering and the dying in past emergencies, give assurance that they have made observations which will promote science and improve the practice of their art.

The whole resources of the revolting States having been required during the late civil war, all the qualified members of the profession friendly to their cause, except those disabled by age or disease, and a limited number imperatively needed at home, voluntarily sought positions in the medical staff of the Confederate armies, and thus strove to fulfill their mission, which is to save and to heal, not to destroy and to wound.

How well they performed their part, time will develop, and history, truly and justly written, will demonstrate. That incompetent and unworthy men intruded themselves among them, the rigid discipline of their branch of the service administered in obedience to their *esprit du corps* undeniably proves. There was no branch of the service in which incompetency was rendered so powerless of harm, and none in which unofficer like conduct was so promptly punished.. We venture to assert that no armies so large were ever supplied with as large a proportion of well qualified surgeons as were those of the South during the recent war. Never, perhaps, was the labor of the surgeons so purely one of love for their patients.

Theirs were not ordinary patients, towards whom indifference could be indulged. Nominally soldiers, they were the neighbors and friends of the surgeons. Selfishly viewed, they were links in the chain of defense, no one of which could be spared. Whatever prejudices may be cherished by a few, growing out of ignorance of the duties and responsibilities of army surgeons, the childishness of the suffering, the distress of fond friends and relatives, and the hardships of conscription, it must be admitted, that in general, the sick and wounded were, by them, most humanely cared for and skillfully treated.

Scantily supplied with exotic remedies, indigenous substitutes were fairly tested, precision and exactness, as well as simplicity in prescription, were encouraged, and the effects of treatment carefully noted.

Rarely were better opportunities afforded for observing the recuperative efforts of nature. Never was human endurance so thoroughly tested. Of the medical topography of the country, of the influence of mental emotions and *morale* upon the physical condition of the soldier, the opportunities for observation were numerous and varied. The frequent changes of station, the varied fortunes of the war, and in many instances the continued service of surviving patients and surgeons, from the beginning to the bitter end, corroborate this assertion.

It is an historical fact that, for the most part, the most active and skillful were stationed at the front, on the battlefield, or in hospitals at convenient distances in the rear, where among the most serious sufferers their services could be commanded. In many instances this character of service was continuously performed during the four years by certain individuals. It is. therefore, no exaggeration to say that there are now living in the prime of manhood among us, surgeons and physicians, whose operative and bedside experience has been rarely, if ever, excelled.

What contributions the surgeons of the late Confederate armies will make to conservative medicine and surgery, remains to be seen. If they are true to their humane instincts and their past history, much is to be expected of them.

May we not hope that they will be permitted to repossess their captured notes and records, that they may collate, arrange and put them into shape for publication in an useful form?

It is the duty of the medical men of the South who have enjoyed such opportunities not to permit private misfortune and the defeat of their cause to deter them from continued exertion in the cause of science and humanity. They must not ignore their obligations. The worthy members of the profession throughout the world have a right to the benefits of their experience. To this end it is desirable that at some early period the members of the profession in the South shall assemble en masse, or through representatives, to devise ways and means of securing a just and truthful history of medicine and surgery in the Confederate armies. Ours is a peaceful calling, and an assemblage of lately rebellious doctors need not excite suspicions of conspiracy against the government in the mind of even the most rabidly radical politician.

So gloomy since the close of the war have been the prospects of pecuniary independence as the reward of the practice of medicine, it is feared that many worthy men have abandoned it. Yet there never was a time when the people of the South were so well prepared to appreciate thoroughly educated medical men.

Such clinical instruction as they have received during four memorable years, has qualified them to appreciate the honest practitioner of medicine.

Circumstances having so long shut out from among them the wares of the quack and nostrum vender, the worthlessness of this species of merchandise is now generally acknowledged.

The people look with more rational confidence to scientific men to instruct them and to treat their diseases. Now, therefore, more than ever, it is in the power of medical men in the South to elevate the standard of professional qualifications. Now, more successfully than heretofore, they can dictate and enforce many needed reforms in medical education.

The people of the South are an earnest people ; they make war in earnest; they make peace in earnest. Whatever calling or pursuit they adopt, they earnestly follow. Soon then, in commerce, shipping, manufactures, mining, the learned professions, they will be competing with the rest of the world for honors and rewards. What may we not hope that clear heads impelled by earnest hearts may achieve in the cultivation of medical science and all the arts of peace ?

Men of education and scientific attainments must arouse to activity. Of what they can accomplish in peace, we have assurance from their conduct during war.

The young now are to be trained. Men and women of accomplished education will not the less be ornaments to society if they assume the task of "teaching the young idea how to shoot," and thereby furnish those facilities, which have too often hitherto been sought abroad.

Amateurs of science must no longer, in the study, selfishly gloat over their intellectual treasures, and, miserlike, be unprofitable members of society. But upon the hills and the mountains, in the valleys and on the waters, they must be found actively employing their talents for useful purposes.

The votaries of medicine propose to appropriate scientific truth to preserve health and relieve human suffering.

To study science and to learn this art is the object, Gentlemen, of your sojourning here.

So honorable did the ancients esteem the medical art, that they denominated it divine, and deified its first professor of whom we have any account.

You live at a time, and among a people, who have, comparatively speaking, divested themselves of superstitious ideas and observances. For now, more than ever heretofore in this Southern land, has the veil of mystery been removed from our art. Men have learned to regard it as based upon scientific truth, and requiring the exercise of strong common sense in its application.

The worthy members of the profession justly make certain demands of you before permitting you to claim a peerage with them. The populace, as before stated, have more adequate and just views than formerly, of the necessary qualifications of a medical adviser.

Among these qualifications are accuracy of information, a well balanced mind, and integrity of character.

Willful or voluntary ignorance is among the worst of crimes, and the student proposing to assume the weighty responsibilities of the physician, that neglects opportunities to qualify himself, is, mayhap, preparing to criminally destroy human lives.

He who cultivates not the judgment, and fails to reason and reflect, is not fit to approach the bedside of human suffering.

He, who with dishonest heart, enters upon the study of medicine, aware that knowledge is power, and with malice prepense, determines to use the knowledge acquired for base and criminal purposes, is an enemy to civilization, to human progress, and to human happiness.

But he, who docile and earnest and honest, with well balanced mind approaches the temple of science, vowing to devote his time and talents to the relief of human suffering and the promotion of human progress, has promised him a rich reward (if not in this world's wealth), in the ineffable pleasure of reflecting, at the end of his career, upon a life well and usefully spent.

Peculiar duties now devolve upon you. You have much to do in preserving the respectability and usefulness of our profession. Great events have recently transpired, in which, perhaps, many of you were energetic actors. In your peaceful calling you may still expect to find ample opportunities for the exercise of generous impulses, and for thought, in advising and co-operating with those around you, who may be laboring to promote social progress and improvement. Your sympathies will find ample opportunities for cultivation in administering to the relief of human suffering.

In the re-opening of the various institutions of learning in the South, you have additional evidence of the recuperative energies of our people.

In appealing to the educated and scientific men of the land, and recalling some of their history, it has been our design to indicate to you in part your duty, and to stimulate you by their example. For the obligations of the student are similar to those of the graduate. With the earnest effort to meet those obligations, you have much to encourage you.

Believing that medicine, in its various branches, can be as well taught in summer as in winter, and in times past, having demonstrated that the dry atmosphere of this city—it being one of the most elevated points of railroad grade between the Gulf and the Tennessee river—favors the perfect preservation of anatomical material throughout the year, knowing that the long days of summer afford many advantages for experimental vivisections, for teaching minor surgery, for demonstrations with the microscope, for clinical instruction, and the exhibition of vegetable materia medica in a growing state, in concurrence with the wishes of many respectable members of the profession, the faculty of this institution have determined to deliver two courses of lectures each year. Thus they propose in eighteen months to give students who desire it, the privilege of attending three courses of lectures instead of only two, as heretofore.

In imposing this additional labor upon themselves, they believe they are acting in accordance with the spirit of the times, which demands that the halls devoted to science shall be always open, and its votaries continuously at work. Henceforward those spacious halls will be open for the benefit of the student throughout the year.

We want no idlers there. In the lecture rooms and in the private class, your teachers expect to work. The wisdom of the policy adopted can only be vindicated by the qualifications and characteristics of the graduates sent forth. Let no one, therefore, suppose that while affording additional facilities, this institution can or shall be used as a means of degrading our profession.

To the faculty a sacred trust is committed, which they can only honorably use to promote science and secure the humane objects of practical medicine and surgery.

They are in earnest when they bid me charge you, who seek the honors of the institution to faithfully do your whole duty, and thus earn the right to claim a respectable position in the ranks of the profession they desire to truly represent.

Amid associations so suggestive of duty, having so many traditions to excite our pride, and honorable examples to imitate without envying the more fortunate, or making invidious comparisons, may we not nerve ourselves to effort, and hope to sustain here an institution of which no scientific man shall be ashamed, and on account of which no Southern man shall have just cause to blush?

Impressed with the responsibilities of the hour, and acknowledging their obligations, while bidding God speed to other honorable enterprises intended to elevate and instruct our people and to diffuse useful knowledge, the guardians of the fair fame of this institution enter upon the labors of the present course, desiring to earn the approbation of their peers, and determined to use every expedient that may tend to ensure thoroughness in teaching, and to adopt that policy which in their opinion is best calculated to elevate the standard of professional excellence. They do not expect to confine their efforts exclusively to teaching by didactic lectures, but by frequent, thorough and searching examinations to stimulate thought and investigation, and to inform themselves thereby of the qualifications of the student at the various stages of his progress in science. This is an age of improvement, and the routine observed by our predecessors of more than half a century ago, is not necessarily binding upon us.

That sickly, not to say silly philanthropy which neglects necessities at home because the sphere of operation is not a whole nation or the whole world, rarely works out useful results. In the home circle is laid the foundation of the happiness of the community. The improvement of communities elevates the nation, and contributes to the aggregate of human progress.

Pretending not that in the South science can be cultivated more successfully than elsewhere, yet denying any inferiority of opportunity on the score of locality, here we find a field for usefulness, promising a fruitful harvest, and affording many stimulants to effort. Our mission as citizens of the world can best be fulfilled by meeting our obligations at home.

If these views are correct, the duty of educated Southern men is plain. They should assist in gathering up the *disjecta membra* of society, and in their respective spheres do their appropriate work in the business of reparation and recuperation. Operating upon and among a people in sympathy with them, whose idiosyncrasies and ethnological peculiarities they understand and appreciate, they can exercise a larger influence for good at home than elsewhere. Cementing the various elements of society by cherishing ennobling impulses, and judiciously encouraging laudable enterprises, they can contribute to the happiness of those around them, while they add largely to the sum of human happiness, and advance the general cause of civilization and human progress.

Animated by such views of duty, the people of the South can retain their identity and self-respect, and work out for themselves a prosperous future.

Let us then to work. Let us not despair.

"Of chance or change, O! let not man complain,
Else shall he never, never cease to wail;
For from the imperial palace, to where the swain
Rears the lone cottage in the silent dale,
All feel the assault of fortune's fickle gale;
Art, empire, earth itself to change are doomed,
Earthquakes have raised to heaven the humble vale
And gulfs the mountain's mighty mass entombed,
And where the Atlantic rolls whole continents have bloomed."

But rather let us courageously buffet waves of present adversity as a means of cherishing a lively hope of future happiness. For

"The man who consecrates his hours,
By vigorous effort and an honest aim;
At once he draws the sting of life and death,
He walks with nature, and her paths are peace."

